# *Lacticaseibacillus paracasei* 36 Mitigates Alcoholic-Associated Liver Disease Through Modulation of Microbiota and AMPK Signaling

**DOI:** 10.3390/nu17142340

**Published:** 2025-07-17

**Authors:** Chongyu Wang, Xi Chen, Fei Wang, Tianyu Chen, Mengqiu Yin, Ziyu Liu, Weifen Li, Jinhui Zhu

**Affiliations:** 1 Department of General Surgery, Second Affiliated Hospital Zhejiang University School of Medicine, Hangzhou 310009, China; 22318533@zju.edu.cn (C.W.);; 2 Key Laboratory of Animal Molecular Nutrition of Education of Ministry, Key Laboratory of Animal Feed and Nutrition of Zhejiang Province, Institute of Animal Nutrition and Feed Sciences, College of Animal Sciences, Zhejiang University, Hangzhou 310058, China

**Keywords:** *Lacticaseibacillus paracasei* 36, ALD, gut microbiota, intestinal barrier, AMPK signaling pathway

## Abstract

Background: Alcohol-associated liver disease (ALD) is characterized by gut–liver axis dysfunction and metabolic dysregulation, yet the therapeutic potential of probiotics remains underexplored. This study aimed to investigate the protective effects and mechanisms of *Lacticaseibacillus paracasei* 36 (LP36) against ethanol-induced ALD in mice. Methods: Mice were pretreated with LP36 prior to ethanol exposure. Liver injury was assessed through serum ALT/AST levels, hepatic steatosis (TC/TG content), and ethanol detoxification capacity (ADH/ALDH activity). Intestinal barrier integrity was evaluated via Mucin2 and ZO-1 expression, and gut microbiota alterations were analyzed by 16S rRNA sequencing. Hepatic transcriptomics (RNA-seq) was performed to identify key regulatory pathways. Results: LP36 significantly attenuated ethanol-induced liver injury, evidenced by reduced ALT/AST, improved hepatic steatosis (lower TC/TG), and enhanced ADH/ALDH activity. Mechanistically, LP36 restored intestinal barrier function (upregulated Mucin2 and ZO-1), modulated gut microbiota (suppressed *Parasutterella*, *Romboutsia*, and *Christensenellaceae_R-7_group*; enriched *Faecalibaculum* and *Tuzzerella*), and reduced systemic inflammation. Transcriptomics revealed LP36-mediated rescue of AMPK signaling, involving regulation of *Stk11*, *Prkag3*, lipid synthesis genes (*Fasn*, *Acaca*), and metabolic modulators (*Creb3l3*, *G6pc3*, *mTOR*, *Rps6kb2*).Conclusions: LP36 ameliorates ethanol-induced ALD by enhancing intestinal barrier integrity, reshaping gut microbiota, and restoring AMPK-dependent metabolic homeostasis. These findings highlight LP36 as a promising probiotic candidate for ALD prevention.

## 1. Introduction

Alcohol-associated liver disease (ALD) represents a major global health challenge, with progressive stages from steatosis to cirrhosis contributing to over 11 million deaths worldwide in 2019 [1]. Current therapeutic strategies primarily focus on promoting ethanol abstinence or late-stage complications of corticosteroids for hepatitis, but fail to address the dysfunction of gut–liver axis that perpetuates ALD progression [2]. Notably, persistent gut microbiota dysbiosis and intestinal barrier leakage aggravate hepatic inflammation through endotoxin translocation, creating an unmet need for interventions aimed at restoring gut homeostasis and correcting metabolic dysfunction [3,4].

Probiotics have garnered significant interest due to their health-promoting properties and potential therapeutic applications in ALD [5,6,7]. By competitively excluding pathogens and enhancing epithelial tight junctions, specific Lactobacillus strains can reduce circulating lipopolysaccharide levels and subsequent Kupffer cell activation [8,9]. Among these, *Lactobacillus paracasei* exhibits unique advantages due to its bile acid-mediated antimicrobial effects and its capacity to produce antioxidant metabolites [10,11]. Preclinical studies in non-alcoholic fatty liver models demonstrate the ability of *Lactobacillus paracasei* to alleviate hepatic lipid accumulation and oxidative stress [12]. A key mechanism bridging gut microbiota modulation and hepatic protection may involve AMP-activated protein kinase (AMPK) [13]. This metabolic sensor regulates lipid oxidation, mitochondrial biogenesis, and inflammasome activation in liver disease [14,15]. It has been proved that probiotics and related products prevent ethanol-induced liver injury by activating hepatic AMPK [16]. *Lactobacillus paracasei* NTU 101 administration enhanced fatty acid oxidation, increasing the expression levels of PPARα, CPT-1, and AMPK proteins in the liver tissue of mice [17], while *Lactobacillus paracasei* supernatants directly activated AMPK in hepatocytes in vitro [18]. LP36 is isolated from Chinese traditional fermented foods [19], and it was found that it demonstrated high capacities for colonization and adhesion, as well as notable antioxidant activity [20], suggesting that LP36 is a promising probiotic to prevent ALD. However, whether this probiotic administration sustains AMPK activation in vivo via microbiota remodeling, and how such crosstalk impacts ethanol-induced liver injury, remains unclear. This study integrates metagenomic, transcriptomic, and functional analyses to establish causal relationships between LP36-induced microbiota change and AMPK-driven hepatic recovery, offering a potential probiotic therapy for ALD.

## 2. Materials and Methods

### 2.1. Bacterial Strains

LP36 was isolated from traditional fermented foods (CCTCC NO M20232287). The cryopreserved strain was initially activated by transferring to MRS broth and incubating statically at 37 °C for 18 h. Single colonies were obtained by streaking on MRS agar plates. A single colony was then inoculated into fresh MRS broth and cultured to mid-log phase. Bacterial cells were harvested by centrifugation (5000× *g*, 5 min), followed by washing three times with PBS (pH 7.4) and removing medium residues. The final pellet was resuspended in sterile PBS, and the density was adjusted to 1 × 10^9^ CFU/mL.

### 2.2. Preparation and Pretreatment of ALD Mice

**Figure 1:**
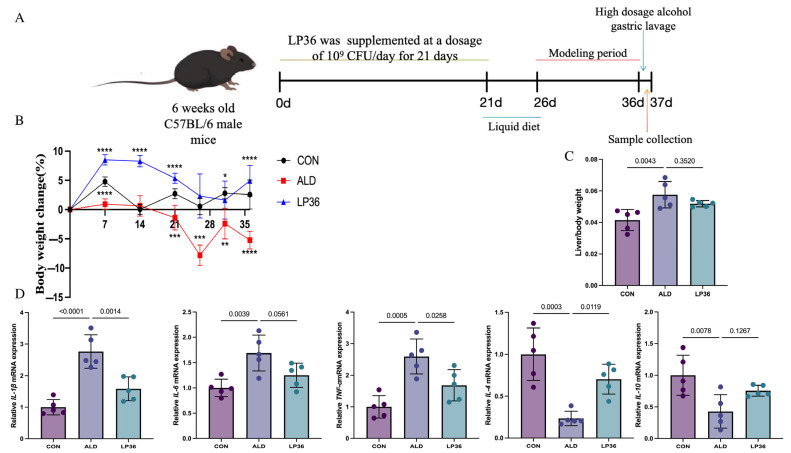
Experimental timeline, body weight change, and effects of LP36 pretreatment in ALD mice. (**A**) Procedure for establishing the ALD mouse model. (**B**) Percentage of body weight variation compared to day 0. (**C**) The ratio of liver body/ weight. (**D**) qPCR analysis of inflammation related genes in the liver. Results are expressed as mean ± SD (*n* = 5 biological replicates per experiment). Statistical significance defined as * *p* < 0.05, ** *p* < 0.01, *** *p* < 0.001, and **** *p* < 0.0001.

As shown in Figure 1A, 18 6-week-old male C57BL/6 mice were randomly divided into the following three groups (6 mice in each group and 3 mice per cage): control (CON, normal diet), alcohol-induced liver disease model (ALD, ethanol challenge), and probiotic intervention (LP36 treatment). After 7 days of acclimatization under specific conditions (22 ± 1 °C, 12/12 h light/dark cycle), pretreatment procedures began. The CON and ALD groups received 200 μL saline daily via oral gavage, whereas the LP36 group was given 200 μL of LP36 suspension (1 × 10^9^ CFU/mL in PBS). After 3 weeks of oral gavage, the NIAAA chronic-binge ethanol feeding model was implemented for 16 days using Lieber–DeCarli liquid diets (Xiaoshu Youtai Biotechnology Co., Ltd., Beijing, China) [21]. On day 16, all mice received intragastric administration at a dosage of 5 g/kg, with the CON group receiving 45% dextrin solution and the experimental group receiving 31.5% (*v*/*v*) alcohol solution. All mice were deeply anesthetized using an ether chamber. Following complete anesthesia, the abdominal cavity was surgically opened, and biological samples (including liver, colon, and cecal contents) were aseptically harvested. Blood was collected via cardiac puncture and centrifuged at 3000× rpm for 15 min at 4 °C to isolate serum. All samples were immediately snap-frozen in liquid nitrogen and stored at −80 °C until further analysis. All animal procedures were approved by the Institutional Animal Care and Use Committee of Second Affliated Hospital of Zhejiang University; the approval number was 2024390.

### 2.3. Histopathological Analysis

Colon and right hepatic lobe tissues were fixed in 4% paraformaldehyde for 24 h, dehydrated through a graded ethanol series, paraffin-embedded, and sectioned at 4 μm. Routine H&E staining was performed, and tissue morphology was evaluated under an optical microscope (40×, random fields). For colon tissues specifically, immunohistochemistry (IHC) was conducted using anti-Mucin2 and anti-ZO1 antibodies (1:200 dilution, Wuhan Servicebio Technology, Wuhan, China). Endogenous peroxidase (3% H_2_O_2_, 20 min) and 5% BSA blocking preceded overnight incubation with primary antibodies (4 °C). HRP-conjugated secondaries and DAB detection were followed by hematoxylin counterstaining. Three random fields/section (Nikon Eclipse E100, 200×, Nikon, Tokyo, Japan) were quantified as IOD using Image-Pro Plus 6.0.

### 2.4. Biochemical Analyses

Plasma levels of alanine transaminase (ALT), aspartate transaminase (AST), triglycerides (TG), and total cholesterol (TC) were quantified using commercially available assay kits (Nanjing Jiancheng Bioengineering Institute, Nanjing, China) according to the manufacturer’s protocols. Simultaneously, hepatic alcohol dehydrogenase (ADH) and aldehyde dehydrogenase (ALDH) activities were determined using a specific enzymatic assay kit (Suzhou Comin Biotechnology Co., Ltd., Suzhou, China).

### 2.5. Quantitative Real-Time Polymerase Chain Reaction (qRT-PCR)

Total RNA of the liver was isolated using RNAiso Plus reagent (Vazyme Biotech Co., Ltd., Nanjing, China). cDNA synthesis and RT-qPCR were conducted with ChamQ SYBR Master Mix (Vazyme) on an ABI 7500 system (Thermo Fisher Scientific, Waltham, MA, USA). Reactions (20 μL) contained 0.4 μM primers (Supplementary Table S1) and β-actin as the endogenous control. Gene expression was calculated via the 2^−ΔΔct^ method.

### 2.6. The 16S rRNA Gene Sequencing and Data Processing

Intestinal content samples were collected from C57BL/6 mice and stored at −80 °C. Total microbial genomic DNA was extracted using the TIANamp Stool DNA Kit (Tiangen Biotech, Beijing, China). The V3–V4 hypervariable regions of the bacterial 16S rRNA gene were amplified with specific primers. Purified amplicons were sequenced on the Illumina MiSeq platform (2 × 300 bp paired-end sequencing) by Novem Metabolomics Co., Ltd. (Hangzhou, China). Raw sequencing data were quality-filtered and processed using QIIME2, followed by amplicon sequence variant (ASV) table generation via DADA2. Beta diversity was visualized through principal coordinate analysis (PCoA) and non-metric multidimensional scaling (NMDS). Shared and unique ASVs across groups were illustrated using Venn diagrams. Alpha diversity indices were computed to assess microbial diversity. Taxonomic classification at phylum and genus levels was performed using the Sliva database.

### 2.7. RNA-Seq Analysis

This study utilized liver tissues from C57BL/6J mice, with transcriptome sequencing and bioinformatics analyses conducted by Novem Metabolomics Co., Ltd. (Hangzhou, China). Total RNA was extracted, and polyadenylated mRNA was enriched using Oligo (dT) magnetic beads. Fragmentation via ion-mediated shearing generated 300 bp fragments, followed by double-stranded cDNA library construction and PCR amplification to select 450 bp products. Quality control was performed using the Agilent 2100 Bioanalyzer (Agilent Technologies, Santa Clara, CA, USA), and paired-end sequencing (PE150) was carried out on the Illumina NovaSeq 6000 platform (Illumina, Inc., San Diego, CA, USA). The mouse reference genome GRCm39 (Ensembl release 108) was employed, containing annotations for 21,959 genes, with comprehensive coverage in GO (98.21%), KEGG (75.22%), and eggNOG (91.22%) databases. Raw reads were filtered for quality, aligned to the genome, and quantified via FPKM. Differentially expressed genes were identified using thresholds of |log2FC| ≥ 1 and FDR < 0.05. Functional enrichment and alternative splicing analyses were subsequently performed.

### 2.8. Statistical Analyses

The relative abundance of microbiota was analyzed using the non-parametric Mann–Whitney U test for pairwise comparisons, while multiple group comparisons were assessed through one-way ANOVA analysis. Statistical significance was determined at *p* < 0.05. All statistical analyses were performed using GraphPad Prism software (v8.0.1; GraphPad Inc., Boston, MA, USA). Quantitative data are expressed as mean ± standard deviation (SD). Statistical significance defined as * *p* < 0.05, ** *p* < 0.01, *** *p* < 0.001, and **** *p* < 0.0001.

## 3. Results

### 3.1. Effects of LP36 on Body Weight Change and Anti-Inflammatory Status in ALD Mice

As shown in Figure 1B, the percentage of weight gain and loss compared to day 0 of the mice were recorded. When fed the same amount of calories, the LP36-treated mice gained more weight than the ALD mice. In Figure 1C, ethanol exposure increased the ratio of liver to body weight, but this effect was slightly reduced by pretreatment with LP36. Finally, inflammation-related genes (*TNF-α*, *IL-1β*, and *IL-6*) were significantly upregulated by ethanol but were reversed by LP36. Meanwhile, anti-inflammation genes, including *IL-4* and *IL-10*, showed the opposite trends.

### 3.2. LP36 Pretreatment Alleviates Ethanol-Induced Liver Injury and Promotes Ethanol Metabolism in Mice

**Figure 2:**
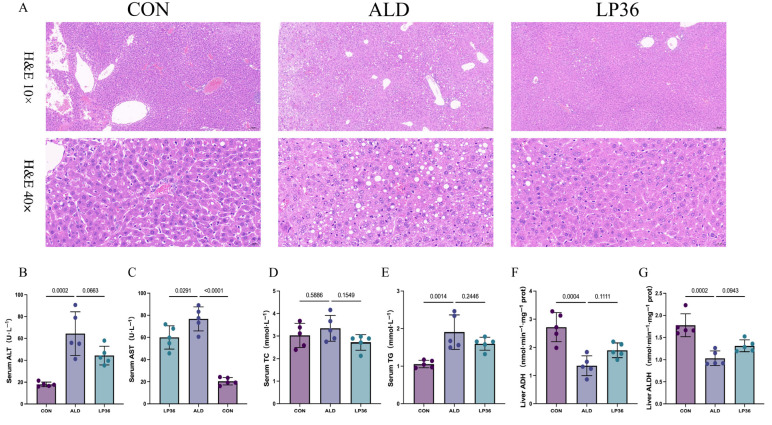
LP36 pretreatment alleviates ethanol-induced liver injury and promotes ethanol metabolism in mice. (**A**) H&E staining. (**B**,**C**) Serum ALT and AST activities. (**D**,**E**) Serum TC and TG levels. (**F**,**G**) Liver alcohol metabolism indicators. The results are presented as mean ± SD (*n* = 5).

According to Figure 2A, there was more severe liver injury and hepatic steatosis, characterized by rounded vacuoles of varying sizes within the cytoplasm, in the ALD mice compared to the mice in the CON group, but milder hepatic steatosis was observed in the LP36 group. To further quantify the liver injury, we measured the serum levels of ALT, AST, TC, and TG in those mice. Similarly, serum ALT and AST activities were elevated by ethanol feeding, but decreased by LP36 pretreatment (*p* = 0.0663 and *p* < 0.05; Figure 2B,C). The levels of TC and TG were significantly elevated in the ALD mice compared to CON, and the LP36 pretreatment resulted in a numerical decrease in both TC (*p* = 0.1519) and TG (*p* = 0.2446) levels compared to the ALD group, although these differences did not reach statistical significance (Figure 2D,E). Finally, we observed that the alcohol metabolism indicators in the liver includes ADH and ALDH, both of which were higher in the LP36 group compared with the ALD group, although these differences did not reach statistical significance (*p* = 0.1111 and *p* = 0.0943; Figure 2F,G).

### 3.3. LP36 Pretreatment Improves Ethanol-Induced Intestinal Injury in Mice

**Figure 3:**
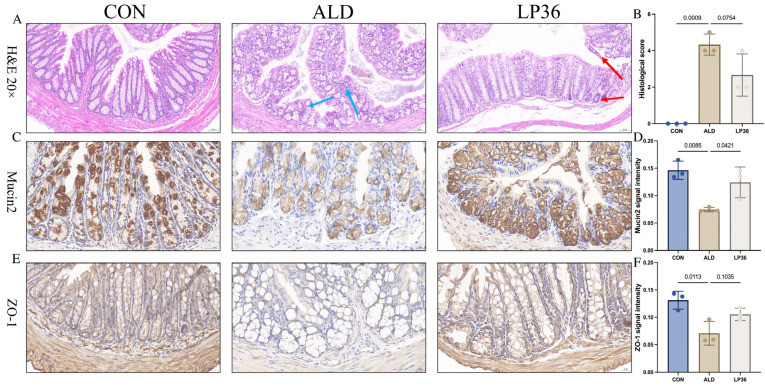
LP36 pretreatment alleviates ethanol-induced intestinal injury in mice. (**A**,**B**) H&E staining and histological score. (**C**,**D**) Relative intensity of Mucin2 in colon. (**E**,**F**) Relative intensity of ZO-1 in colon. The results are presented as mean ± SD (*n* = 3). (**C**,**E**) scale bar = 20 µm.

Alcohol exposure induced colonic histopathological changes, featuring glandular atrophy with reduced and disorganized goblet cells, mucosal thinning, heterogeneous muscularis thickening, and focal inflammation, which were improved by the LP36 pretreatment (Figure 3A,B). To further analyze the function of the intestinal barrier, we performed IHC of Mucin2 and ZO-1 in the colon. As shown in Figure 3C,D, the relative intensity of Mucin2 in ALD mice was lower than that in the CON group, but there was significantly higher intensity of Mucin2 observed in the LP36 mice compared to the ALD group. Meanwhile, there was a higher intensity of ZO-1 in the LP36 mice, with no significant difference compared to the ALD group (*p* = 0.1035, Figure 3E,F).

### 3.4. LP36 Improves the Gut Microbiota Composition in Mice

**Figure 4:**
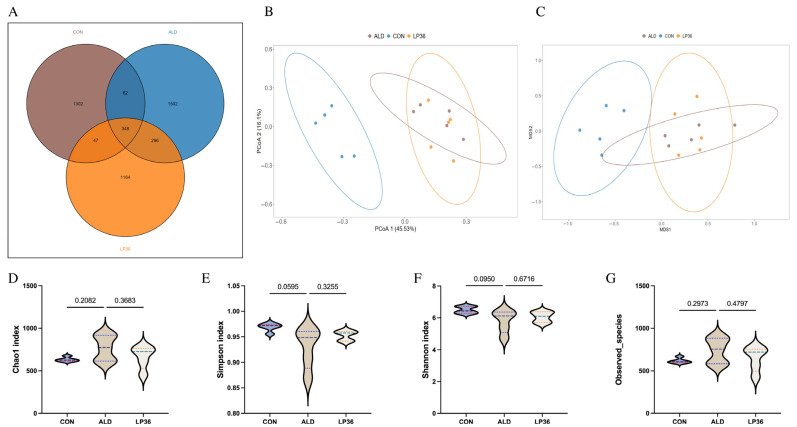
LP36 improves the gut microbiota composition in mice. (**A**) Venn diagram. (**B**,**C**) PCoA and NMDS analysis. (**D**–**G**) α diversity analysis. The results are presented as mean ± SD (*n* = 5).

**Figure 5:**
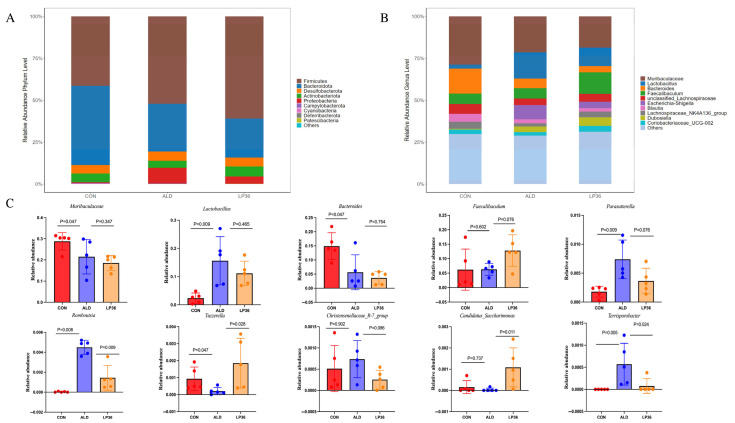
LP36 improves the gut microbiota composition in mice. (**A**) The relative abundance at the phylum level. (**B**) The relative abundance at the genus level. (**C**) The main genera affected by LP36. The results are presented as mean ± SD (*n* = 5).

As it is well known that the microbial environment influences intestinal barrier function, we further analyzed changes in the gut microbiota composition. A Venn diagram showed that there were 348 OTUs overlapping in all groups, and 1302 OTUs, 1502 OTUs, and 1164 OTUs in the CON group, ALD group, and LP36 group, respectively (Figure 4A). According to Figure 4B,C, there were no significant differences in the β diversity among all groups. Ethanol treatment tended to decrease the microbial abundance compared to the CON group (*p* < 0.1), but there was no significant difference (*p* > 0.05) between the ALD and LP36 groups for α diversity (Figure 4D–G). To further characterize differential microbial taxa and explore the taxonomic composition, we assessed species profiles at both the phylum and genus levels. As is shown in Figure 5A–C, pretreating with LP36 elevated the relative abundance of *Faecalibaculum*, *Tuzzerella*, and *Candidatus_Saccharimonas*, and decreased the relative abundance of *Parasutterella*, *Romboutsia*, *Christensenellaceae_R-7_group*, and *Terrisporobacter* compared to the ALD group.

### 3.5. LP36 Regulates the Liver Transcriptomics in Mice

**Figure 6:**
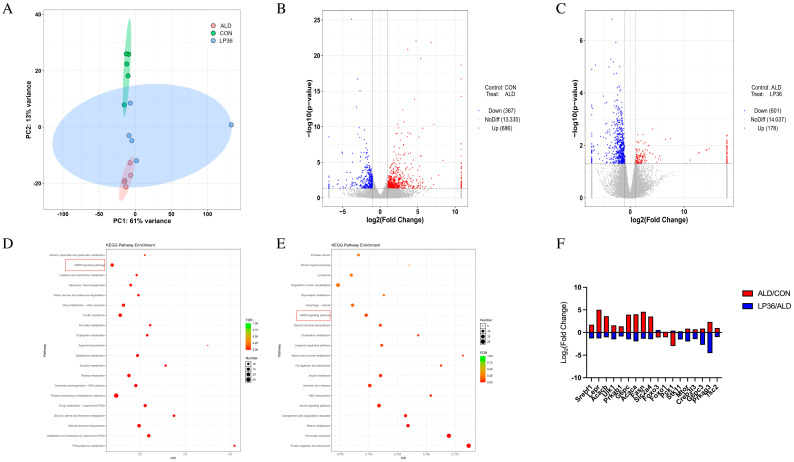
LP36 regulates the liver transcriptomics in mice. (**A**) PCA analysis. (**B**,**C**) Volcano map. (**D**,**E**) KEGG enrichment analysis. (**F**) The log2FC of AMPK-related genes.

**Figure 7:**
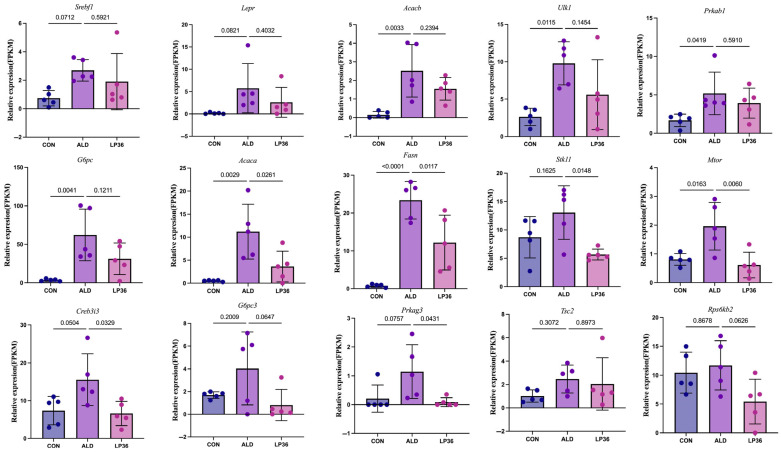
FPKM values of differentially expressed AMPK-related genes. The results are presented as mean ± SD (*n* = 5).

**Figure 8:**
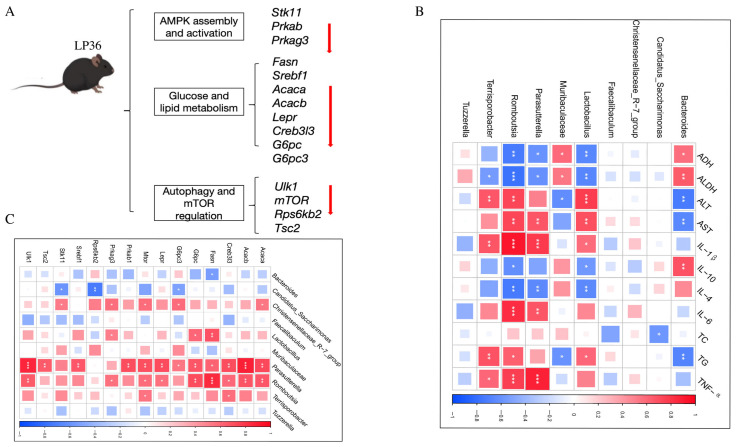
Function of genes and correlation analysis. (**A**) Function of differentially expressed AMPK-related genes. (**B**) Correlation between the gut microbiota and liver injury and inflammation parameters. (**C**) Correlation between the gut microbiota and AMPK-related genes. Statistical significance defined as * *p* < 0.05, ** *p* < 0.01, and *** *p* < 0.001.

To investigate the molecular basis of hepatic functional alterations, transcriptomic profiling was performed. PCA analysis demonstrated distinct clustering of the CON, ALD, and LP36 groups, indicating significant inter-group transcriptional divergence (Figure 6A). Comparative analysis revealed 1053 ethanol-induced DEGs (686 upregulated and 367 downregulated in ALD vs. CON) and 779 LP36-modulated DEGs (178 upregulated and 601 downregulated in LP36 vs. ALD) (Figure 6B,C). KEGG pathway enrichment identified the AMPK signaling pathway as a central regulatory mechanism (Figure 6D,E). Quantitative analysis of log2FC and FPKM values identified ethanol-driven transcriptional activation of AMPK signaling components (*Stk11* and *Prkag3*), lipid synthesis regulators (*Fasn* and *Acaca*), and metabolic modulators (*Creb3l3*, *G6pc3*, *mTOR*, and *Rps6kb2*), all of which were normalized by LP36 intervention (Figure 6F, Figure 7 and Figure 8A).

### 3.6. Correlation Among Liver Injury and Inflammatory Parameters, Gut Microbiota, and AMPK-Related Genes

To elucidate the gut microbiota–liver axis interplay, we systematically analyzed correlations between the gut microbiota composition, hepatic AMPK-related genes, liver injury, and inflammatory indices. Notably, following the LP36 pretreatment, the relative abundance of *Candidatus_Saccharimonas* was significantly increased and exhibited a negative correlation with the TC level. In contrast, the LP36 pretreatment reduced *Parasutterella*, *Romboutsia*, and *Terrisporobacter*, which demonstrated distinct associations with metabolic, inflammatory, and hepatic indices. These genera were negatively correlated with ADH and ALDH; meanwhile, they displayed positive correlations with markers of hepatic injury, including AST and ALT, as well as the TG level. Furthermore, these taxa showed the following divergent relationships with cytokine profiles: negative correlations were observed with anti-inflammatory cytokines IL-4 and IL-10, while positive correlations were detected with pro-inflammatory mediators, including IL-6, IL-1β, and TNF-α (Figure 8B). As shown in Figure 8C, the abundance of *Candidatus_Saccharimonas*, which was significantly increased by the LP36 pretreatment, showed negative correlations with genes involved in gluconeogenesis *(G6pc3*), mTOR signaling (*Rps6kb2*), and energy-sensing pathways (*Stk11*). Conversely, the taxa reduced by the LP36 pretreatment, including *Parasutterella*, *Romboutsia*, and *Terrisporobacter*, exhibited negative correlations with key regulators of lipid and glucose metabolism. These included *Acaca* and *Acacb* (acetyl-CoA carboxylases and fatty acid synthesis), *Fasn*, and *Srebf1*. Additionally, these genera were inversely associated with *Lepr* and *G6pc*, critical for energy homeostasis, as well as *Creb3l3* (metabolic stress regulator). Intriguingly, they displayed positive correlations with autophagy-related genes (Ulk1) and negative correlations with AMPK/mTOR pathway components (*Prkab1*, *Prkag3*, *Tsc2*, and *Mtor*), suggesting disrupted coordination between nutrient sensing and catabolic processes.

## 4. Discussion

Alcohol-related liver disease (ALD), a prevalent complication of chronic alcohol consumption, represents a significant global health burden owing to the liver’s crucial role in xenobiotic detoxification and lipid homeostasis [22,23]. In addition to conventional therapeutic approaches, including abstinence, hepatoprotective agents, and physical activity, accumulating evidence supports the potential utility of probiotics as therapeutic adjuvants for alcohol-induced hepatic disorders [24,25,26,27]. In the present study, the probiotic LP36 demonstrated significant efficacy in attenuating ethanol-induced hepatic dysfunction and lipid accumulation in ALD models, as indicated by reduced serum TG/TC/AST/ALT levels, enhanced liver ADH/ALDH activities, and the downregulation of inflammation-related gene expression. These observations are consistent with previous reports documenting probiotic efficacy in ALD management [28,29]. Mechanistic investigations suggest that LP36 may confer hepatoprotection through multiple pathways: the modulation of the gut microbiota composition, the enhancement of intestinal epithelial barrier integrity, and the regulation of hepatic AMPK-related gene transcription.

Emerging evidence underscores the pivotal role of ethanol-induced gut microbiota dysbiosis in the pathogenesis of ALD [30,31]. Probiotic interventions have demonstrated therapeutic potential through multiple mechanisms, including microbiota stabilization, intestinal barrier reinforcement, and systemic anti-inflammatory effects [32,33,34]. Among various probiotic strains, specific Lactobacillus species have shown particular promise for ALD management via microbiota modulation. Notably, Ben Niu’s research group demonstrated that *Lactobacillus paracasei* supplementation effectively restored gut microbial homeostasis, enhanced intestinal barrier integrity, and attenuated ethanol-induced hepatic injury through the activation of antioxidant pathways [9]. Supporting these findings, a separate clinical investigation reported that the administration of *Lactobacillus paracasei* LP N1115 improved clinical outcomes in hepatic encephalopathy patients by modifying the gut microbiota composition and reducing systemic inflammation [35]. These consistent observations across preclinical and clinical studies establish microbiota modulation as a viable therapeutic paradigm for alcohol-related pathologies. Consistent with previous reports, we also found that alcohol exposure markedly elevated *Parasutterella*, *Romboutsia*, and *Terrisporobacter*, which were effectively suppressed by LP36. *Parasutterella*, a bile acid metabolism-associated genus, has been reported to increase in dysbiotic states, including metabolic disorders and liver diseases, where its overabundance may correlate with disrupted bile acid homeostasis and inflammation [36,37]. The observed reduction in *Romboutsia* (*Clostridiaceae* family) may indicate a transition toward an anti-inflammatory gut milieu. While *Romboutsia* exhibits context-dependent roles, its enrichment in ALD models correlates with gut barrier impairment and increased endotoxin translocation [38]. Similarly, the decreased *Parasutterella* abundance suggests microbial rebalancing that may ameliorate bile acid dysregulation, a known contributor to ALD progression [39]. LP36-driven suppression of *Romboutsia* might mitigate intestinal permeability, thereby reducing hepatic exposure to bacterial endotoxins such as lipopolysaccharide, a key driver of ALD pathogenesis [40,41]. Notably, the reduction in *Terrisporobacter*, a genus known for spore formation and associations with opportunistic infections [42], further supports the potential of LP36 to counteract ALD-related dysbiosis. Elevated *Terrisporobacter* levels have been documented in inflammatory bowel disease, and that *Terrisporobacter* can affect TG and HDL-C has been confirmed by Lee SH et al. [43,44], suggesting its role in exacerbating gut-derived inflammation and the imbalance of lipid metabolism, and the decrease in this genus could thus contribute to ameliorating systemic inflammation and metabolic disturbances in ALD. Thus, our findings indicate that LP36 could improve the microbiota composition in ALD mice, endorsing the colonization of beneficial bacteria, and inhibiting the colonization of potentially pathogenic bacteria.

Chronic alcohol consumption is well established to impair intestinal barrier integrity via multiple pathological mechanisms [45]. The small intestine, functioning as a dynamic transitional interface, facilitates microbial translocation toward distal segments through rapid luminal fluid flow before significant bacterial proliferation occurs. This anatomical region, together with the colon, constitutes a crucial ecological niche for colonization by exogenous Lactobacillus strains [46]. Prolonged ethanol exposure induces both structural and functional compromise of the intestinal epithelium, manifesting as the reduced expression of tight junction proteins (including Claudin-1 and ZO-1) and increased intestinal permeability [47,48]. In our study, prophylactic LP36 administration exhibited significant protective effects against alcohol-induced barrier dysfunction, as demonstrated by the upregulated Muc2 and ZO-1 expression. These results corroborate previous findings reporting comparable barrier-protective properties of probiotic strains such as *Lactobacillus rhamnosus* GG and *Lactobacillus paracasei* Jlus66 in alcohol-related gut injury models [49,50]. Collectively, these observations reinforce the therapeutic potential of specific lactobacilli in mitigating ethanol-induced intestinal epithelial damage through the modulation of mucin synthesis and the stabilization of junctional complexes.

The correlation analysis between the gut microbiota composition and markers of liver injury and inflammation reveals that LP36 pretreatment modulates gut microbiota by enriching *Candidatus_Saccharimonas* (negatively correlated with TC) and reducing *Parasutterella*, *Romboutsia*, and *Terrisporobacter*. These suppressed taxa showed negative associations with ADH and ALDH but positive links to hepatic injury markers (AST, ALT, and TG) and pro-inflammatory cytokines (IL-6, IL-1β, and TNF-α), alongside inverse correlations with anti-inflammatory IL-4 and IL-10. This suggests that LP36 alleviates ALD by promoting beneficial taxa like *Candidatus_Saccharimonas* to improve lipid metabolism, while curbing harmful genera that exacerbate inflammation, impair detoxification, and drive hepatic damage. The microbiota shifts induced by LP36 may thus restore gut–liver axis balance, highlighting its therapeutic potential in mitigating alcohol-related metabolic and inflammatory dysregulation.

This study demonstrates that LP36 alleviates ethanol-induced hepatic metabolic disturbances, likely through the modulation of the AMPK signaling pathway. AMP-activated protein kinase (AMPK), a central regulator of cellular energy homeostasis, plays a pivotal role in modulating lipid metabolism, glucose utilization, and inflammatory responses [51]. Ethanol exposure triggered the significant upregulation of AMPK-related genes (*Stk11*, *Prkab*, and *Prkag3*), reflecting the energy stress and altered AMP/ATP ratios during alcohol metabolism [52]. Recent studies have identified AMPK as a key mediator of probiotic effects, reporting that *Lactiplantibacillus plantarum* B19 enhances hepatic insulin sensitivity via AMPK/PI3K/AKT pathway activation [53], and that the *Lactobacillus rhamnosus* GG supernatant restores AMPK phosphorylation to mitigate alcohol-induced hepatic lipid dysregulation and apoptosis [54]. These findings underscore the potential of probiotic interventions to modulate AMPK signaling in liver disease. LP36 intervention normalized these expression patterns, suggesting the restoration of AMPK activity and metabolic equilibrium. Concurrently, ethanol activated lipid synthesis genes (*Fasn*, *Srebf1*, *Acaca*, and *Acacb*), which LP36 effectively suppressed, thereby aligning with AMPK’s role in inhibiting acetyl-CoA carboxylase (*Acaca*) and suppressing sterol regulatory element-binding protein 1 (*Srebf1*) to curb fatty acid and cholesterol synthesis [55]. LP36 also restored dysregulated metabolic regulators (Lepr and Creb3l3) and autophagy-associated genes (*Ulk1*, *mTOR*, and *Tsc2*) potentially via AMPK-mTOR crosstalk, where AMPK activation inhibits mTORC1 to promote the autophagic clearance of lipid droplets. While these findings highlight the capacity of LP36 to rebalance hepatic metabolism through AMPK-driven mechanisms, further work is needed to clarify whether its effects are mediated by direct microbial metabolites or indirect pathways involving gut–liver axis communication.

The gut–liver axis mediates interactions through gut microbiota-derived metabolites, immune mediators, and microbial signals, which collectively regulate hepatic metabolism, inflammatory responses, and functional integrity, serving as a key determinant of physiological equilibrium and disease susceptibility [56]. These microbial components exert dynamic control over hepatic gene expression via epigenetic remodeling, nuclear receptor signaling, and inflammatory pathway modulation, directly linking gut-derived signals to liver diseases [57,58]. Further analysis highlighted microbial interactions with the following key metabolic genes: *Fasn* and *mTOR* showed strong positive correlations with *Parasutterella* and *Romboutsia*, while *Srebf1* and *Tsc2* were exclusively linked to *Parasutterella*. These findings collectively underscore the gut microbiota’s potential modulation of hepatic AMPK signaling, lipid metabolism, and inflammatory cascades, implicating specific taxa as microbial influencers of metabolic dysregulation or resilience.

## 5. Limitations

While this study demonstrates the protective effects of LP36 against ALD, several limitations should be acknowledged. First, although transcriptomic analysis revealed that LP36 modulated AMPK signaling-related genes, the lack of protein-level validation limits the mechanistic interpretation of AMPK activation. Future studies should incorporate both mRNA and protein expression analyses to confirm these findings. Second, 16S rRNA sequencing identified LP36-induced gut microbiota alterations, but metabolomic profiling was not performed to link microbial changes to functional outcomes. Such data could provide deeper insights into how LP36-improved microbiota contribute to hepatic AMPK regulation by a metabolizer. Third, the study was conducted in a mouse model, and human trials are needed to validate LP36’s efficacy in ALD patients.

## 6. Conclusions

This study demonstrates that *Lacticaseibacillus paracasei* 36 (LP36) effectively protects against ethanol-induced liver injury by improving gut barrier function, modulating gut microbiota, and restoring metabolic balance. LP36 reduces hepatic steatosis, enhances alcohol detoxification, and activates AMPK signaling to alleviate liver damage. These findings highlight LP36 as a promising probiotic for preventing and treating alcohol-associated liver disease.

## Data Availability

The original contributions presented in this study are included in the article/Supplementary Material. Further inquiries can be directed to the corresponding authors.

## Supplementary Materials

The following supporting information can be downloaded at: https://www.mdpi.com/article/10.3390/nu17142340/s1. Table S1: Primer sequences for qRT-PCR.
