# Supplementary material for: Lacticaseibacillus paracasei 36 Mitigates Alcoholic-Associated Liver Disease Through Modulation of Microbiota and AMPK Signaling
Source: Nutrients. 2025 Jul 17;17(14):2340. doi: 10.3390/nu17142340 (PMC12298492; doi:10.3390/nu17142340)
Supplement: Supplementary file 1 [file nutrients-17-02340-s001.zip › nutrients-3689092-supplementary.pdf]

Supplementary Table S1 Primer sequences for qRT-PCR

Name	Sequence
TNF-α	F: 5’-CCCTCACACTCACAAACCAC-3’
	R: 5’-ACAAGGTACAACCCATCGGC-3’
IL-1β	F: 5’-TGCCACCTTTTGACAGTGATG-3’
	R: 5’-ATGTGCTGCTGCGAGATTTG-3’
IL-6	F: 5’-ACAAAGCCAGAGTCCTTCAGAG-3’
	R: 5’-TGTGACTCCAGCTTATCTCTTGG-3’
IL-4	F: 5’-TCACTGACGGCACAGAGCTA-3’
	R: 5’-TTTGGCACATCCATCTCCGT-3’
IL-10	F: 5’-ACCTGGTAGAAGTGATGCCC-3’
	R: 5’-TGTAGACACCTTGGTCTTGGA-3’
β-actin	F: 5’-GACTACCTCATGAAGATCCTGACC-3’
	R: 5’-TCGAAGTCTAGAGCAACATAGCAC-3’
